# Health-related quality of life and survival of cancer patients admitted to ICUs: Results of the QALY study

**DOI:** 10.1186/cc11018

**Published:** 2012-03-20

**Authors:** AB Cavalcanti, UV Silva, KN Normílio-Silva, AN Silva, R Zancani, MJ Giorgi, AD Dias, AT Simone, PL Safra, AC Figueiredo, G Tunes-da-Silva, AC Lima, LA Hajjar, JO Auler, J Eluf-Neto, FR Galas

**Affiliations:** 1 São Paulo State Cancer Institute, São Paulo, Brazil; 2 Barretos Cancer Hospital, Barretos, Brazil; 3 Instituto de Matemática e Estatística - Universidade de São Paulo, Brazil; 4 Faculdade de Medicina da Universidade de São Paulo, Brazil

## Introduction

Very limited data are available regarding postdischarge health-related quality of life (HRQL) of cancer patients needing intensive care. Our objective is to describe HRQL and survival in an unselected population of cancer patients who were admitted to ICUs.

## Methods

In this prospective cohort study conducted at two cancer hospitals in Brazil, we enrolled a random sample of adult patients with cancer admitted to the ICUs. We collected data at ICU admission, including HRQL before the acute process that led to ICU admission, and followed patients up on 15, 90 and 180 days after ICU admission to assess HRQL and vital status. We determined HRQL with the EQ-5D questionnaire, and the results were presented as summary measures with values between -1 and 1, with 0 meaning HRQL similar to death and 1 perfect HRQL. Summary measures were calculated using time-trade-off value sets obtained from the UK population. Survival was calculated with the Kaplan-Meier estimator.

## Results

We enrolled 805 patients. Mean age was 61.4 ± 14.3 and 42.5% were female. Elective surgeries represented 52.2% of admissions, urgent surgeries represented 5.0% and 42.8% were admitted due to clinical reasons. Survival at 180 days was 51.2% (95% CI 47.4 to 54.9). The HRQL summary measure (median (interquartile range)) before ICU admission was 0.64 (0.12 to 0.81), on the 15-day follow-up 0.73 (0.19 to 0.92), on the 90-day follow-up 0.73 (0.20 to 0.85) and on the 180-day follow-up 0.70 (0.35 to 0.89).

## Conclusion

HRQL is, on average, moderately impaired before ICU admission and through the 180-day follow-up in cancer patients needing intensive care. Only about one-half of the patients were alive after 180 days. However, there is large variability on both HRQL and length of survival; thus, methods to estimate quality-adjusted life-years on an individual basis are necessary.

